# Development and usability evaluation of an electronic health report form to assess health in young people: a mixed-methods approach

**DOI:** 10.1186/s12911-023-02191-7

**Published:** 2023-05-10

**Authors:** Petra V Lostelius, Magdalena Mattebo, Eva Thors Adolfsson, Anne Söderlund, Mikael Andersén, Sofia Vadlin, Åsa Revenäs

**Affiliations:** 1 Clinic for Pain Rehabilitation Västmanland, Region Västmanland, Västerås, Sweden; 2 School of Health, Care and Social Welfare, Mälardalen University, Västerås, Sweden; 3 Centre for Innovation, Research and Education, Region Västmanland, Vastmanland Hospital, Vasteras, Sweden; 4 Centre for Clinical Research, Region Västmanland – Uppsala University, Region Vastmanland, Vasteras, Sweden; 5 Department of Women’s and Children’s Health, Uppsala University, Uppsala, Sweden; 6 Orthopedic Clinic, Västerås hospital Region Västmanland, Västerås, Sweden

**Keywords:** Developmental study, Electronic patient-reported outcome, Mixed methods research, Usability study, Medical informatics, Participatory research, Young people, Youth health clinic

## Abstract

**Background:**

Electronic Patient-Reported Outcomes (ePROs) have potential to improve health outcomes and healthcare. The development of health-technology applications, such as ePROs, should include the potential users and be theoretically grounded. Swedish Youth Health Clinics (YHCs) offer primarily sexual and psychological healthcare for young people aged 12 to 25 years old. Young people in healthcare settings are considered a vulnerable group. The development of a collection of Patient-Reported Outcomes (PROs) in an Electronic Health Report Form (eHRF) for identifying health and health-related problems in young people, was preceded by a qualitative interview study, exploring young people’s views on using an eHRF at YHCs and which questions about health an eHRF should contain. The aim of the current study was to develop and evaluate the usability of an eHRF prototype for identifying health and health-related problems in young people visiting YHCs.

**Methods:**

This study used a participatory design. During the development, an expert panel consisting of eight researchers and one Information Technology worker, participated. A wide literature search was performed to find PROs to construct an eHRF prototype to cover health areas. A mixed methods usability evaluation included 14 participants (young people, healthcare professionals, and an expert panel).

**Results:**

The development resulted in an eHRF prototype, containing ten reliable and valid health questionnaires addressing mental-, physical-, and sexual health and social support, a self-efficacy question, and background questions, in total 74 items. The interviews in the usability evaluation resulted in three categories describing the usability of the eHRF: ‘Captures the overall health of young people but needs clarification’, ‘Fun, easy, and optional and will keep young people’s interest’, and ‘Potential contribution to improve the health consultation’. The quantitative results support the usability of the eHRF for YHCs.

**Conclusions:**

The participatory approach contributed to development of the eHRF prototype to cover health areas adapted for the target population. The usability evaluation showed that the eHRF was usable and had the potential for self-reflection and contributions to cooperation between young people and healthcare professionals during the health consultation.

**Supplementary Information:**

The online version contains supplementary material available at 10.1186/s12911-023-02191-7.

## Introduction

Health technology, such as Electronic Patient-Reported Outcomes (ePRO), have advantages over paper-based Patient-Reported Outcomes (PRO), for example decrease costs, improve data quality, and facilitate clinical symptom management and decision making [1, 2]. The use of ePROs has the potential to improve health outcomes [3], and influence patients and organizations, such as healthcare services, to work toward personalized health-related needs [4]. Both ePROs and analog PROs provide evidence-based [3], self-reported patient health status [5], and help to understand the treatment impact on functioning and well-being [6]. The development of health-technology applications, such as ePROs, demands an extensive process characterized by the participation of technology developers, clinicians, and patients to ensure that relevant health outcomes are captured [7–9], the technology fits within the healthcare systems, and is adequate and durable for the users [3].Validation of ePROs may be important, depending on the degree of modifications made when converting from PROs to ePROs [2].

Participatory research is used to develop applications for assessing health and health-related problems in young populations. In New Zealand, the ePRO YouthCHAT [10], is used for assessing psychosocial health (mental health and risky behaviors) in young people from 13 to 25 years of age [10, 11], long-term physical conditions at outpatient clinics [12] and Maori youth [13], as well as investigating the motivation for accepting help [14]. In the context of a young Swedish population (12 to 25 years) visiting Youth Health Clinics (YHCs), the SEXual health Identification Tool (SEXIT), is used to identify young people at increased risk of, or with existing poor sexual health [15], and the young person’s will to address health-related issues. Staff at YHCs have found SEXIT useful in YHC health assessments [16]. YHCs in Sweden focus on strengthening sexual and reproductive health and the rights of youth and young adults, but also address young people’s physical and mental well-being. Young people are a vulnerable group, as they often lack of autonomy [17] in the healthcare setting. For example, young people may find it hard to ask about health concerns and vulnerable feelings to healthcare professionals. This can lead to health risks not being detected by healthcare, which contributes to health inequity [18].

Human health is complex and depends on an interaction of biological, psychological, and social factors, as described by the biopsychosocial theory [19]. This is also reflected in the World Health Organization’s (WHO) 1948 definition of health as “a state of complete physical, mental, and social well-being, not merely the absence of disease or infirmity” (page 1) [20]. Thus, the assessment with ePROs used at YHCs needs to consider not only sexual health, but also other aspects of young people´s health. A previous interview study with fifteen young people, aged 17 to 22 years, visiting five YHCs in two regions in central Sweden showed that an electronic assessment for young people should include questions about mental-, physical-, sexual health and social support, and have the potential for self-reflection and increased self-awareness [21].

With the previous study as a starting point, the aim of this study was twofold. First, to develop a collection of ePRO questionnaires; an Electronic Health Report Form (eHRF) prototype. The eHRF prototype should contain ePRO questionnaires in four health areas, to identify health and health-related problems in young people visiting YHCs. Second, the aim was to evaluate the usability of the eHRF prototype.

## Materials and methods

The development of the eHRF prototype was based on the theoretical foundation [22] from a participatory research approach [23]. For the current study, participation means the right of young people and healthcare professionals to influence decisions for the content and layout of an eHRF for the YHC [24]. The development of an eHRF prototype was guided by biopsychosocial theory [19] and a holistic view of health. The concept of self-efficacy [25] was used to clarify the state of engagement for possible behavior change.

The study was approved by the Regional Ethics Committee, Uppsala, Sweden (dnr 2020 − 01921) and was performed according to the principles of the Declaration of Helsinki [26].

### Study design

This study was conducted during autumn 2020 and consisted of two parts: (I) the development and (II) the usability evaluation of the eHRF prototype.

**Fig. 1:**
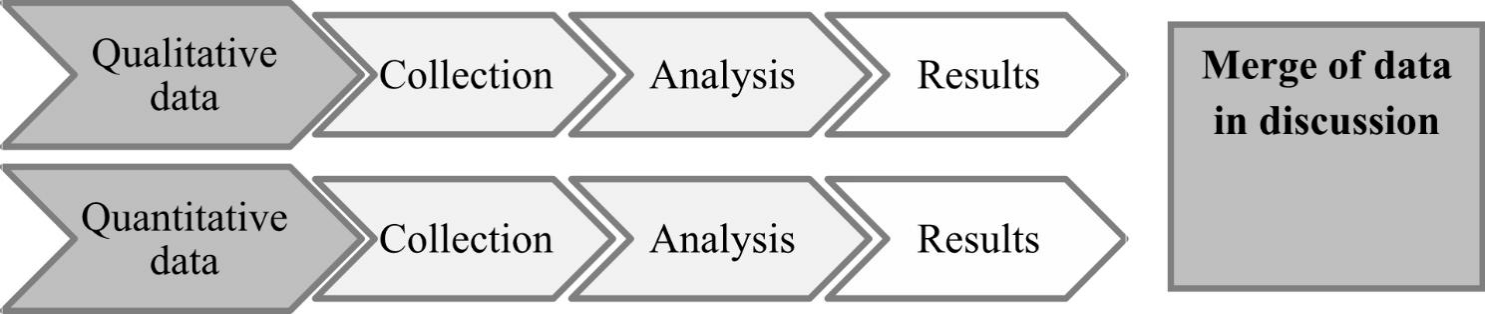
Mixed-methods convergent design in the usability evaluation of the Electronic Health Report Form (eHRF) prototype

I) To develop an eHRF prototype for identifying health and health-related problems in young people visiting YHCs, a wide literature search was performed, based on a previous interview study [21].
II) The usability evaluation of the eHRF prototype was applied with a mixed-methods convergent design, using qualitative and quantitative data collection in a side-by-side approach and merging of data by discussion [27], as shown in Fig. 1.

### Participants

Overall, a diverse group of participant characteristics was wanted, to include as many aspects of knowledge, expertise, and experience as possible, which is important in participation research [28]. Young people had previously participated in identifying the different health areas to include in an eHRF for YHC [21], allowing the researchers’ perspective to assert itself in the development phase. The target number of participants for the development phase was an expert panel with eight researchers. For the usability evaluation, the participant target number was 12 young people and three YHC healthcare professionals.

#### eHRF prototype development participants

**Fig. 2:**
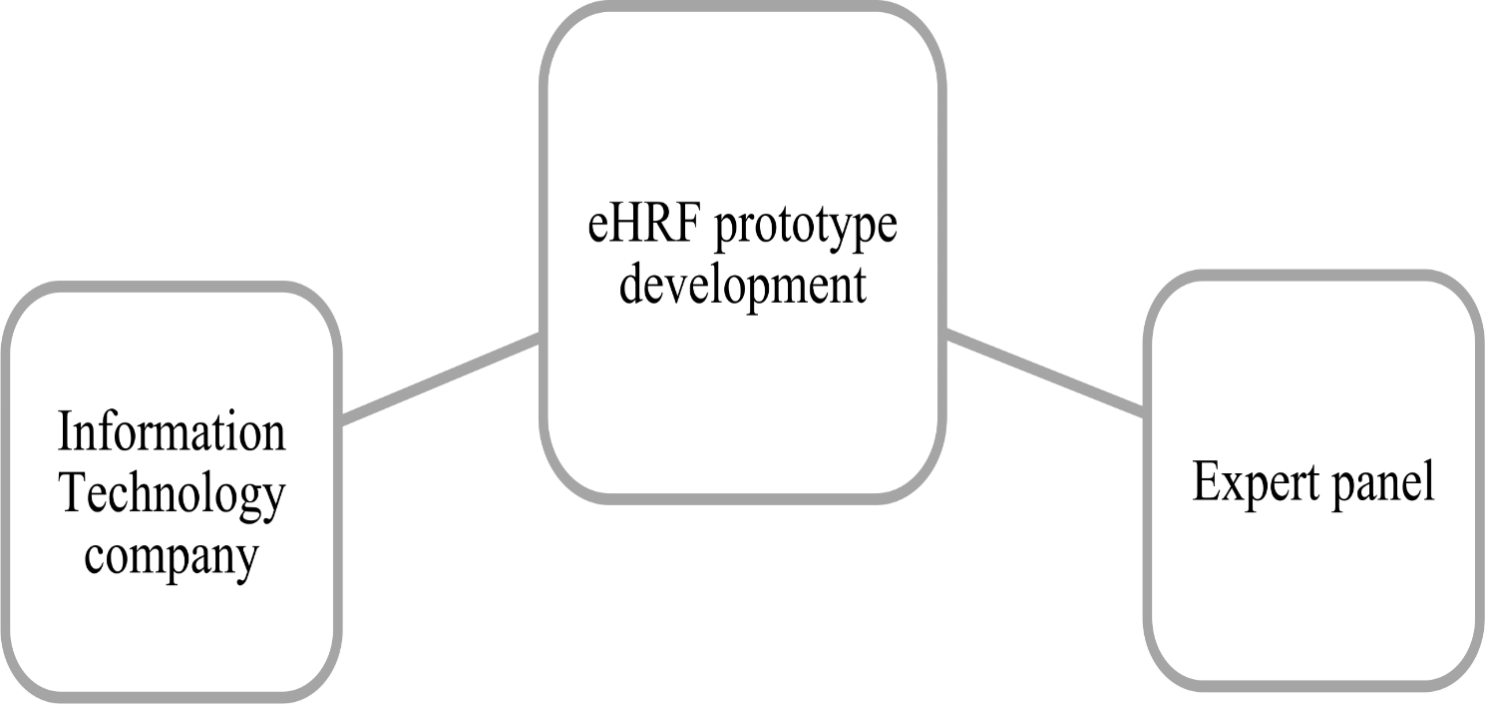
The participants in the Electronic Health Report Form (eHRF) prototype development

Participants in the development of the eHRF prototype were an expert panel and an Information Technology (IT) company (Fig. 2).

The expert panel members were purposively selected based on their professional and academic experience. The participating members had multi-professional clinical experience in the development of health technology and working with young people in mental healthcare, primary healthcare, and YHCs. Their academic degrees ranged from Master of Science to professor.

A collaboration with an IT-company contact person was included. The IT-company had experience in collaboration with several universities and healthcare regions in Sweden, offering solutions for a medical documentation system [29].

#### eHRF prototype usability evaluation participants

**Fig. 3:**
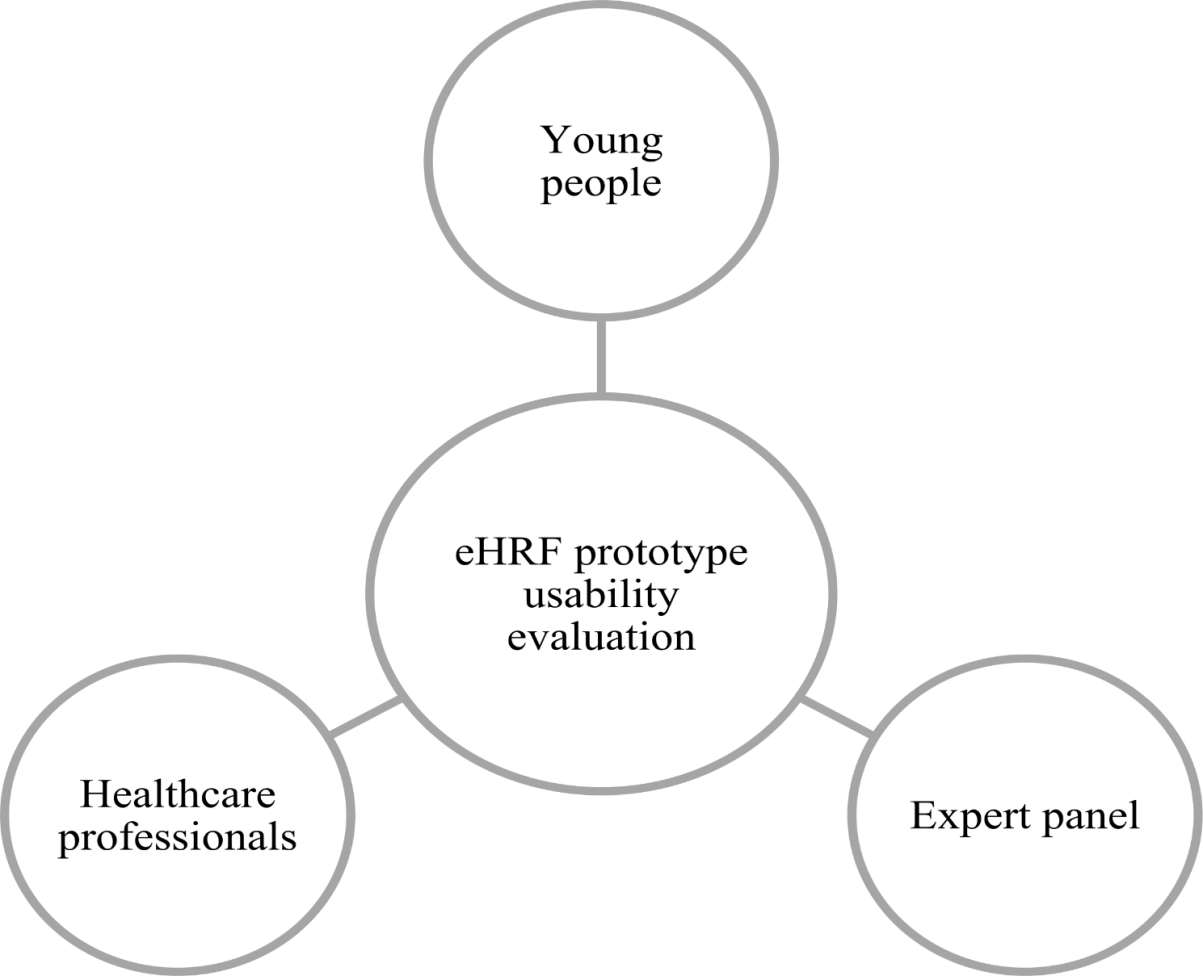
The participants in the Electronic Health Report Form (eHRF) prototype usability evaluation

Participants in the eHRF prototype usability evaluation were young people, healthcare professionals and an expert panel (Fig. 3). A convenient sample of healthcare professionals and young people were included from the same selected YHC, situated in a small municipality in Central Sweden, taking approximately 25 appointments per week. The expert panel were affiliated with five regions in Central Sweden.

Inclusion criteria were: 16–23 years old, visiting the YHC, and being fully fluent in Swedish. Heterogeneity was desired in terms of gender identity, place of birth, living conditions, sexual orientation, and level of education. The young people were informed about the study by healthcare professionals during a planned visit to the YHC. They were asked about participation and to be contacted by the first author (PVL) for information on voluntary participation and confidentiality, registration in the IT system, and time for data collection.

All healthcare professionals working at the selected YHC participated in the study and received information about the study in an e-mail that included information on voluntary participation and confidentiality, and registration in the IT system. Appointments were booked for the data collection.

The purposively selected expert panel members were chosen using the same criteria used for the eHRF prototype development expert panel members. The experts were e-mailed information about the study and invited to a digital group meeting.

### eHRF prototype development

#### Data collection

**Fig. 4:**
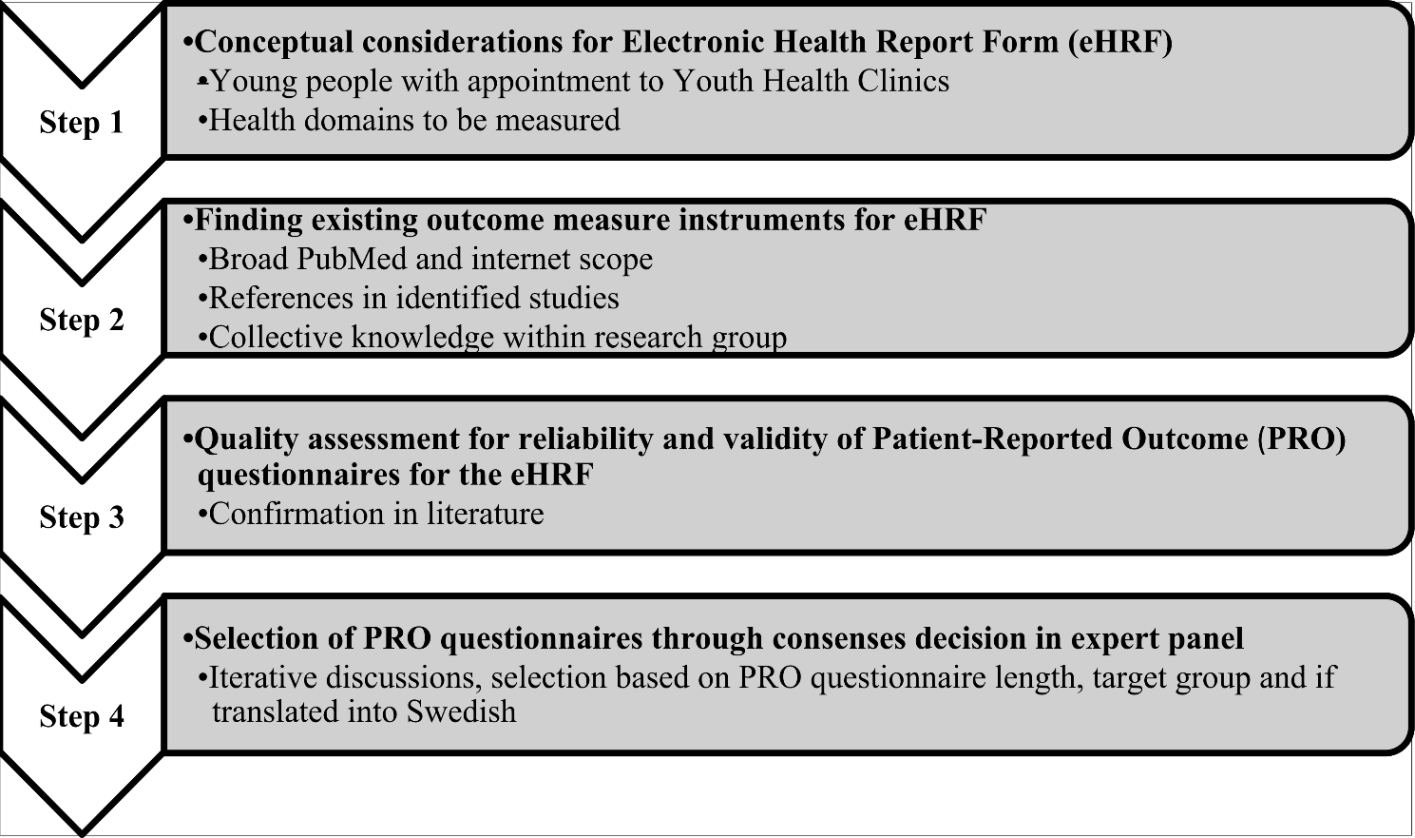
Development process with COnsensus-based Standards for the selection of health Measurement Instruments (COSMIN) guidelines

The selection of PRO questionnaires for the eHRF prototype was in line with the COnsensus-based Standards for the selection of health Measurement Instruments (COSMIN) guidelines for selecting outcome measurements [30], presented in Fig. 4. A stepwise process started with determining the domains to be measured. Consensus for the health areas to focus on was reached within the research group, based on the previous interview study with young people [21]; physical health (including lifestyle habits), mental health (including questions about self-harm and suicide risk), sexual health and sexual experience and relationships/social support. The next step involved finding the PROs to include. This was based on a broad PubMed scope and internet search, performed by PVL in 2019. The search involved keywords and phrases, for instance, “health”, “health questionnaire”, eHealth, and “young people/youth/adolescent”. The Swedish website https://www.fbanken.se, a resource targeted at healthcare professionals and constructed to collect valid health questionnaires, primarily concerning mental health and social support, was also consulted for appropriate questionnaires. The references in all identified studies were explored. The expert panel’s clinical work and research experience contributed with suggestions for questionnaires to, and ideas for searching for new questionnaires to consider for the eHRF prototype.

#### Analysis

An iterative analysis process took place amongst members of the expert panel. The panel discussed appropriate questionnaires for the eHRF prototype, based on validity and reliability. The PRO questionnaires were evaluated for reliability and validity, questionnaire length, target group and if translated into Swedish.

The iterative process helped to limit the selection of included PRO questionnaires and ended when consensus had been reached among the expert panel.

#### eHRF prototype construction

The selected PRO questionnaires were compiled in a paper version. The researchers PVL and AS constructed one question of self-efficacy for change in accordance with Bandura’s [31] work, with the recommended 100-poing Numerical Rating Scale (NRS) for responses.

To prepare the questionnaires for digitalization, PVL, ETA and MA coded the questionnaire items and response options in Microsoft Excel. The IT-company digitalized the questionnaires in close collaboration with PVL and ETA. The eHRF prototype was divided into sections based on content: physical health (including lifestyle habits), mental health, sexual health, and social support. Each PRO questionnaire started with the visual aid of an emoji, portraying its’ content. The emojis were all free to use from https://pixabay.com/sv/images/search/emojies/.

The digitalization process and the eHRF prototype functionality were tested by the expert panel in an iterative process and changes were made continuously until coherence was reached.

### eHRF prototype usability evaluation

#### Data collection

##### Interviews

All participants were informed of the study conditions and gave consent before the interviews. The interviews were either in person at the YHC or digital and were performed individually except for the expert panel who participated in a digital group interview. Participants in digital interviews gained access to the eHRF through an SMS to their smartphones. Participants in face-to-face interviews gained access to a tablet, provided by PVL. The interviews followed a semi-structured interview guide (Table 1). PVL performed the interviews, which lasted 50–90 min. All interviews were recorded with an external voice recorder and transcribed verbatim. For details in qualitative data collection procedure, see Supplementary file 1.

**Table 1:** The interview guide for the usability aspects of the Electronic Health Report Form (eHRF) prototype

Topic	Main questions	Follow-up questions
The eHRF ability for identification of health	What is your opinion of the ability of the eHRF prototype to capture young people’s health?	How do you find the health content of the eHRF prototype? Is there any health area missing and if so, which?
The eHRF prototype structure	What is your opinion of the structure of the eHRF prototype?	How do you find the layout of the eHRF prototype regarding the position of questions? What is your opinion of the design of the eHRF prototype, for example, the health sections, emojis? How do you perceive, for instance, the question about behavioral change or social support?
The eHRF prototype functionality	What is your opinion of how to use the eHRF prototype at the Youth Health Clinics (YHC)	What do you believe that the eHRF prototype can mean for the visit at the YHC?
Final question	Is there anything else you want to add to the interview?	

##### System Usability Scale

The System Usability Scale (SUS) [32] is a 10-item scale (Table 2) applied to assess the users’ perceived usability of a product [33]. A five-point Likert scale, ranging from Totally disagree [1] to Totally agree [5], was used for each item. The Swedish version SUS 1,4_sv [34] was used. The scale’s original phrase “system” has been changed to “eHRF prototype”.

The SUS scores can be ranged and converted to different grades of usability [33], to help interpretation of the scores. Values from 71.4 to 100 are viewed as “good usability” up to “best imaginable usability”. Values of 80 or more are considered above the average usability mean score [33].

**Table 2:** The statements of the System Usability Scale questionnaire [34] with indications of negative or positive statements

	Statement	positive	negative
1	I think that I would like to use the eHRF prototype.	X	
2	I found the eHRF prototype unnecessarily complex.		X
3	I thought the eHRF prototype was easy to use.	X	
4	I think that I would need the support of a technical person to be able to use this eHRF prototype.		X
5	I found the various functions in this eHRF prototype were clear and well organized.	X	
6	I thought there were too many contradictions and illogical pathways in this eHRF prototype.		X
7	I would imagine that most people would learn to use this eHRF prototype very quickly.	X	
8	I found the eHRF prototype very awkward to use.		X
9	I felt very confident using the eHRF prototype.	X	
10	I needed to learn a lot of things before I could get going with this eHRF prototype.		X

Notes: Electronic Health Report Form prototype (eHRF prototype)

### Data analysis

Inductive qualitative content analysis was performed for the interviews [35]. All transcribed interviews were read repeatedly for familiarization with the data. Throughout the process, dialog occurred between the researchers (PVL, ÅR, MM, ETA, AS). First, two transcripts were read and meaning units were coded separately by two of the authors (ÅR and PVL) with the purpose of ensuring agreement on the process and content of important aspects of the data. Comparison was made to strengthen credibility before proceeding with the analysis. Data describing the participants’ views on usability and ideas for improvements to the eHRF prototype were coded. The development of the categories was discussed and elaborated several times between the authors to establish equal content within the category and orthogonality between the categories (Table 3).

**Table 3:** Examples of the schematic analysis process

Transcription	Condensation	Code	Sub-category	Category
I think all … important parts were there … everything from … well sexual issues are usually… why young people kind of go there … but there are other things as well. Like violence and relationships and stuff like that. I think it was a great mix of everything.	I think all parts were there, sexual issues but other things as well. It was a great mix of everything.	Good mix of questions from different health areas.	Can identify the right health areas, appropriate for young people.	Captures overall health of young people but needs clarification.
It wasn’t just like … a boring survey, all white and black and … with black text and just very boring … it had a little more feeling.	It wasn’t just a boring survey.	Not boring.	Needs an easy-going and natural design.	Fun, easy and optional will keep young people’s interest.

The SUS Likert scale scores for each item and participant will be presented and converted into a total value for each participant according to instructions [33].

## Results

### Participants

Totally participated, in the eHRF prototype development, eight researchers with variety of academic experiences in the expert panel (Table 4) and one participant represented the IT-company.

**Table 4:** Characteristics of the expert panel participants

Gender		Profession	Academic degree
Female		Midwife	Associate professor
Female		Dietician	Associate professor
Male		Psychologist	Master of Science
Female		Healthcare counselor	Researcher
Female		Physiotherapist*	Doctoral student
Female		Physiotherapist	Associate professor
Female		Physiotherapist	Professor
Female		Nurse	Associate professor

In total 14 people participated in the eHRF prototype usability evaluation. Young people (n = 4) and healthcare professionals (n = 3) participated in both the qualitative and quantitative usability evaluation. Demographic information is presented in (Tables 5 and 6). The members of the expert panel, except the doctoral student (n = 7), participated in the qualitative usability evaluation.

**Table 5:** Demographic information of the young people

Age	Housing	Lives with	Place of birth	Sexual orientation	Level of education
16–20	Villa	M, F, S	Sweden	Heterosexual	High school
16–20	Villa	Alternately with M (new partner), S and F	Sweden	Heterosexual	High school
16–20	Rental apartment	Alone	Sweden	Heterosexual	High school
16–20	Villa	M, F, S	Sweden	Heterosexual	High school

Notes: Mother (M), Father (F), Sibling (S), Sweden (Swe)

**Table 6:** Demographic information of the healthcare professionals from the Youth Health Clinic (YHC).

Age	Profession	Years at YHC
30–35	Healthcare counselor	1
55–60	Midwife	11
55–60	Manager	19

**Fig. 5:**
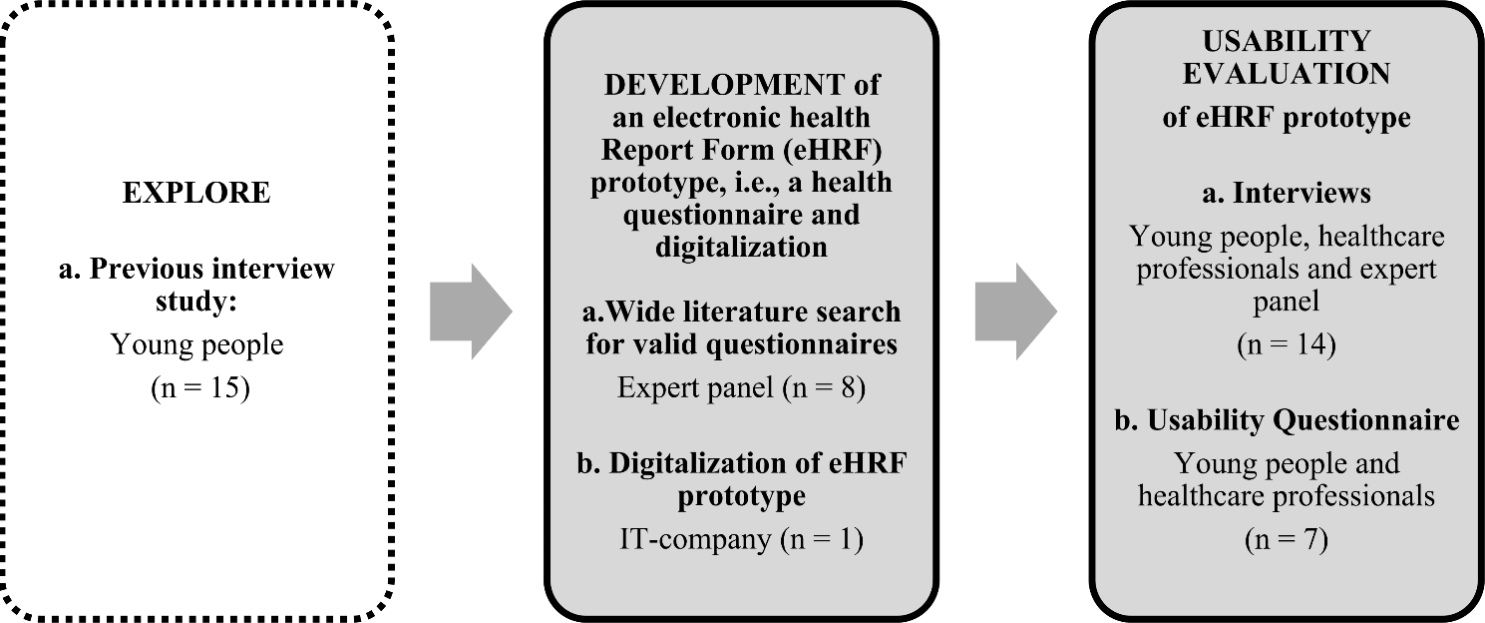
Process and participants in developing and evaluating the Electronic Health Report Form (eHRF) prototype [21]

The participants in the development and usability evaluation of the eHRF prototype are displayed in Fig. 5.

### The eHRF prototype development

A total of 43 health questionnaires (17 physical health, 19 mental health, 3 sexual health, 4 social support) were identified for possible inclusion in the eHRF prototype. The iterative analysis process contributed to the selection of 10 questionnaires: consisting of 66 items, and seven background questions. The questionnaires the SEXual health Identification Tool (SEXIT) [15], the questions for the National Guidelines: Living Habits [36], and the questions for the National Guidelines: Living Habits, indicator questions for physical activity [36, 37] and the study-specific self-efficacy question were developed in Swedish [31] (for English versions, see Supplementary file 2. The questionnaires Alcohol Use Disorders Identification Test – Consumption (Audit-C) [38], Health Behavior in School-aged Children (HBSC) [39, 40], Generalized Anxiety Disorder 7-item scale (GAD-7) [41], Patient Health Questionnaire-9 (PHQ-9) [42], The SCOFF Questionnaire (acronym from the questions) [43], and Berlin Social Support Scales (BSSS) subscales “Need for support” and “Support seeking” [44] were available in Swedish (for English version see Supplementary file 2). The selected questionnaires had at least acceptable reliability and validity in its original language. However, some questionnaires were not tested for the YHC age group 12 to 23.

In total, the eHRF prototype consisted of 74 items (Table 7).

**Table 7:** The Electronic Health Report Form (eHRF) prototype with background questions, reliability, and validity

Item	Health area	questionnaire	Reliability/validity
	A. Background questions		
A1	Reason for visiting Youth Health Clinics (YHC)		
A2–A4	Age, Gender, Sex	SEXual health Identification Tool (SEXIT) (15)	Content- and face validity, acceptability for YHCs age group. Swedish (4).
A5 A6	Sexual orientation Living conditions	SEXIT (15) SEXIT (15)	
A7	Ongoing healthcare contact outside YHC		
	B. Physical health		
B1–B4	Alcohol, drugs, and tobacco	Alcohol Use Disorders Identification Test – Consumption (Audit-C) (38), SEXIT (15), National Guidelines: Living Habits (36)	Audit-C: Best choice for harmful and high-volume drinking. Swedish [59]. National Guidelines: Living Habits. Swedish (36): Used nationally and recommended for healthcare.
B5–B9	Nutrition and eating habits	National Guidelines: Living Habits (36)	Used nationally and recommended for healthcare.
B10–B11	Physical activity and sedentary time	National Guidelines: Living Habits, indicator questions for physical activity (36, 37)	Used nationally and recommended for healthcare.
B12	Behavior change	Self-efficacy Scale (31)	
B13–B18	Bodily symptoms	Health Behavior in School-aged Children (HBSC) (40)	Adequate validity and satisfactory test-retest reliability (40). School-age children. Swedish.
	C. Mental health		
C1–C8	Anxiety/worry	Generalized Anxiety Disorder 7-item scale (GAD-7) (41)	Good reliability and criterion-, construct-, factorial- and procedural validity from age 18 (41). Swedish.
C9–C18	Sadness/depression	Patient Health Questionnaire-9 (PHQ-9) (42)	Validity and reliability acceptable from age 18 (42). Swedish.
C19–C23	Relation to food and body	The SCOFF Questionnaire* (43)	Acceptable validity for adolescents, especially girls [60]. Swedish.
	D. Experience of violence		
D1–D9	Experience of violence	SEXIT (15)	Content- and face validity, acceptability for YHC age group. Swedish (4).
	E. Sexual health		
E1–E10	Sex habits	SEXIT (15)	Content- and face validity, acceptability for YHCs, Swedish (4).
	F. Social support		
F1–F6	Social support	Berlin Social Support Scales (BSSS) subscales “Need for support” and “Support seeking” (44)	Initial validation for adult cancer patients (44), used many contexts. Not available in Swedish
F7	Behavior change	The Bandura Self-efficacy Scale (31)	Constructed in accordance with Bandura’s guidelines of how to construct self-efficacy scales (31).
Total	74 items		

*SCOFF = acronym from the five questions included in the SCOFF questionnaire [43], based on a person’s relationship to food and body

One PRO questionnaire, The Berlin Social Support Scale (BSSS) [44] was not previously translated to Swedish. Therefore, a translation process was performed. PVL and AS translated the English version of BSSS. The Swedish translation was sent to an American psychologist/researcher, fluent and living in Sweden, who translated the BSSS back to English. PVL and AS reviewed the translation and only made adjustments to a few items to improve comprehension in Swedish.

### The eHRF prototype usability evaluation

#### Interviews

**Fig. 6:**
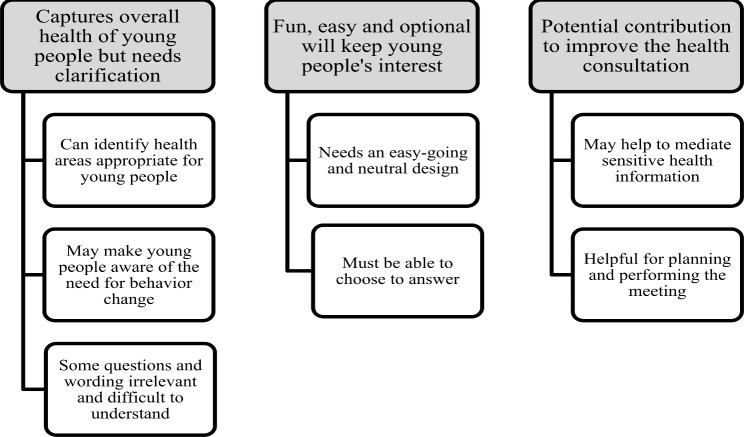
Overview of the categories and subcategories for the Electronic Health Report Form (eHRF) prototype usability

The qualitative content analysis of the transcribed interviews resulted in three categories and seven subcategories. The categories are described below and displayed in Fig. 6. Throughout the results section the term “participants” is used when young participants, healthcare professionals, and the expert panel are included. A more detailed description of the categories and subcategories including citations is available (Supplementary file 3).

#### Captures overall health of young people but needs clarification

This category confirmed that the eHRF prototype health areas (mental-, physical-, and sexual health and social support) captured the overall health of young people. The young participants believed that the eHRF prototype had the potential to make young people aware of their health and possible need for behavior change. However, for young people who were not willing to change, the self-efficacy question of behavior change could be difficult to answer.

There were suggestions for improvements, for example remove questions on hight and weight and neutralize gender descriptions. Also, they suggested to focus more on family relations. The participants wanted a definition for “social support” and the term “behavioral change” changed to “lifestyle change” and a need for another word for “anxious”. The healthcare professionals found the self-efficacy behavior change question important and connected it to their experience of the challenge of having conversations about behavior change.

#### Fun, easy and optional will keep young people’s interest

This category stated that an easy-going and neutral eHRF prototype design, good layout, and structure could help young people answer the health questions. Although they liked the emojies in the eHRF, they suggested that the emojis could possibly be exchanged for other pictures or even colors or fonts. The expert panel and healthcare professionals, on the other hand, were hesitant or critical toward details in the layout, suggesting that the emojis used in the eHRF prototype could affect young people by portraying an emotion about the health area and questions it represented; they wanted the emojis to be changed to something more neutral, commenting that “less is more.” The participants found it essential that young people could choose to answer the health questions before the meeting. Both young participants and healthcare professionals wanted young people to be able to disregard questions that they were uncomfortable answering.

#### Potential contribution to improving the health consultation

The third category summarized that the eHRF prototype was found to bring something valuable to the health assessment conversation. The young participants thought that the eHRF would help them to answer honestly to sensitive questions. The healthcare professionals, on the other hand, hesitated on the questions’ sensitive topics and suspected that young people may not even complete the eHRF prototype questions. They also suspected that answering questions without knowing who they would meet at the YHC could make young people unwilling to use the eHRF prototype.

The young participants believed that answering the health questions could make them more focused on their health and help them prioritize which health areas that were more important. The healthcare professionals agreed with the young participants and said that the eHRF prototype questions and the young people´s answers could form solid ground for talking with young people about health and contributing to their professional evaluation.

### The satisfaction usability scale

The individual and the sum of participants’ scores for each item of the SUS are shown in Tables 8 and 9. The participants claimed high agreement with all statements. The participants rated highly, the positive statements “I think I would like to use the eHRF prototype” and I would imagine that most people would learn to use this eHRF prototype very quickly”. They disagreed most with the negative statement “I think that I would need the support of a technical person to be able to use this eHRF prototype”. There were no missing items in the SUS responses.

**Table 8:** The participants individual scores and sum for each positive statement of the System Usability Scale

Odd items	1 Like to use	3 Easy to use	5 Clear & organized	7 Quick to learn	9 Confident using it
Participants					
1	4	4	5	5	5
2	5	5	5	5	5
3	5	4	5	5	5
4	5	5	4	5	5
5	4	4	3	3	4
6	5	5	5	5	3,5
7	5	3	3	5	5
Total score	**33/35**	**30/35**	**30/35**	**33/35**	**32,5/35**

Notes: The total score per item ranged from 7–35, with higher scores reflecting more optimal usability. Participants 1–3 were healthcare professionals and participants 4–7 were young people

**Table 9:** The participants individual scores and sum for each negative statement of the System Usability Scale

Even items	2 Too Complex	4 Need support	6 Too many contradictions	8 Awkward to use it	10 Needed to learn before
Participants					
1	1	1	1	2	1
2	1	1	1	1	1
3	1	1	1	2	1
4	1	1	1	1	1
5	1	1	2	3	2
6	1	2	1	1	2,5
7	3	1	3	2	1
Total score	9/7	8/7	10/7	12/7	9.5/7

Notes: The total score per item ranged from 7–35, with lower scores reflecting more optimal usability. Participants 1–3 were healthcare professionals and participants 4–7 were young people

**Fig. 7:**
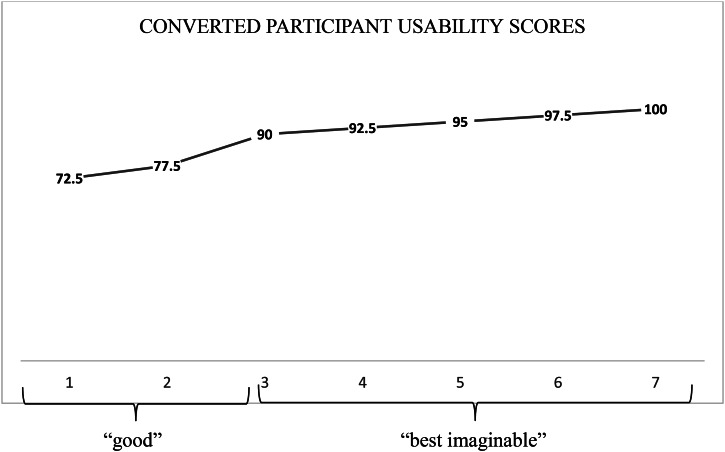
Participants’ (n = 7) individually converted usability scores and corresponding grades.

The SUS scores were converted to grades of usability [33]. The converted scores show that five participants’ scores concurred with the grade “best imaginable” (participants 3, 4, 5, 6, 7) and two participants’ scores were concurrent with the grade “good” (participants 1 and 2), as shown in Fig. 7.

## Discussion

This study had a participatory research approach to develop and evaluate an eHRF prototype with credible content and usability at YHCs. The current study was based on a previous interview study with young people at YHCs [21] that was the start of the development process, exploring of content and design of an eHRF for YHCs and hence, strengthening the young person’s perspective in this study. Due to ethical considerations, the youngest YHC visitors (12–15 years) were excluded. However, the participants in the eHRF evaluation represent the clinical YHC young- and healthcare professional population. Together with the expert panel participants, the group shows diversity in ages, knowledge, and experiences.

The search for and selection of PRO questionnaires in this development of the eHRF were mostly in line with the COSMIN guidelines [30]. However, the literature search had limitations in structure and search terms, reducing the possibility to reproduce the search. The construction was strengthened by the previous interview study of young people [21] and the expert panel’s experience of health questionnaires. Including several stakeholders [45], and performing the development process in steps [30], are ways to assess content validity, described by Almanasreh, Moles and Chen [46]. The researchers developed the eHRF prototype from ten questionnaires [28, 33–41], in total 74 items including background questions and a self-efficacy question. Self-efficacy questions have potential to increase the understanding of young individuals’ ability to change behavior to improve health [25], and occur in PROs/ePROs for young people.

The evaluation of the eHRF prototype usability consisted of qualitative interviews and a quantitative usability questionnaire. The combination of the qualitative and quantitative data was assumed to provide additional perspectives and a more complete understanding [27] of the usability of the eHRF prototype. Overall, the results indicated that the eHRF prototype was usable for YHCs. In summary, there were positive correlations between qualitative and quantitative data, indicating that the eHRF prototype was easy to understand and found easy to use. The qualitative data showed that the young participants were also positive toward the eHRF prototype possibility to increase their understanding of health. This is supported by the previous interview study, stating that answering meaningful health questions in an eHRF prototype could potentially lead to self-reflection and increased self-awareness [21]. Kutcher et al. [47] has highlighted the importance of increasing mental health literacy, i.e., how well individuals can understand, and communicate about health-related information for making informed health decisions (page 16) [48]. Same-level health conversations between young person and healthcare professional may act as the starting point for behavior change to improve health because it involves self-reflection as well as information for the consultation with the healthcare provider that will help determine suitable interventions [49]. In young people, health technology, has been used to identify psychosocial issues [11–13] and improving health equity [50]. Additionally, young people have pointed out the importance of electronic health questionnaires for time-efficient health assessments, directed toward what is essential to the young person [51].

The young participants thought that responding to health questions before meeting with a healthcare professional could make it easier to honestly answer sensitive questions. This has found also, by Thabrew et al. [52]. However, the healthcare professionals in the current study were concerned that the questions on sensitive topics may make it hard for young people to answer honestly. This was also found in a qualitative study that interviewed healthcare professionals at YHCs for their experiences of using SEXIT [16]. This may implicate a need for training healthcare professionals to talk about sensitive topics.

The qualitative findings highlight the importance of an appealing design, to keep young people’s interest and help them complete the eHRF prototype health questions, also supported by the previous interview study [21]. In the current study the layout and design of the eHRF prototype was appreciated by the young people, and the emojis viewed as refreshing and fun. This has been found important to improve usability and user satisfaction in guiding clinical decisions [53]. However, healthcare professionals and expert panel disagreed to some of the emojies.

The study had several limitations. The target number of participants for the usability evaluation was not reached. There were in total fourteen participants in the usability evaluation (four young people, three healthcare professionals, seven members of the expert panel). The few young people included reflects that the estimated number of appointments were even lower, due to the COVID-19 pandemic restrictions, and the water leak causing poor environment at the YHC. Another study limitation was that only seven participants responded to the SUS questionnaire. For a significant outcome, the SUS questionnaire requires at least eight participants [33]. However, for early usability evaluations, five participants have been found sufficient to identify usability issues [54]. An additional weakness is that all participants were female. Although a study limitation, this is reflective of the YHC patient population, supported by surveys, showing that almost 90% of YHC visitors are female [55]. All considered, population in the current study should be satisfactory for the usability evaluation in the YHC setting. Another limitation was that inconsistent records were kept of the young people who declined to participate, due to the clinical pre-requisites at the YHC. Finally, the ongoing COVID-19 pandemic during the study period demanded adaptions to the study procedure, for example digital interviews. This may have affected the candor of the participants’ responses to the SUS.

This study acknowledges that research needs to serve the society and its current context [56]. Hence, it was pragmatically designed to allow clinical and research solutions and to involve the future users, i.e., young people and healthcare professionals. The eHRF developed in this study, consisting of several PROs (traditionally non-digital), has not been validated. However, no changes were made to the PROs when converted digitally, indicating that the need for validation is lower [2]. Also, ePROs provide better data quality, decrease costs, and facilitate clinical symptom management and decision-making compared to PROs [1]. Still, if implementing the eHRF prototype for clinical use at YHCs, future research is needed to validate the eHRF prototype for ages 12–15 and for other healthcare settings. This eHRF has potential to provide a structured and unified assessment of young people’s health. This may reduce the risk for health inequities among young people [57].

The next research step is to further develop the eHRF prototype in considering the improvement suggestions from the participants, for example removing some questions and exchanging emojies. After improving the eHRF, a feasibility study (protocol ISRCTN23855544) is planned to evaluate feasibility aspects (process, resources and management), [58], before performing a fourth-coming Stepped Wedge Cluster Randomized Trial (SW-CRT).

## Conclusions

This study presents the results from the development and early usability testing of an eHRF using a participatory approach. The collaboration of an expert panel and an IT company resulted in the development of an eHRF prototype. It was based prior findings of young people’s opinions on important health areas and valid and reliable PRO questionnaires. The eHRF reflects the biopsychosocial perspective, including self-efficacy for behavior change as a shared base for a health conversation between healthcare professional and young people at YHCs. The usability evaluation showed that the eHRF prototype was usable, could lead to self-reflection and cooperation between young people and healthcare professionals during the health consultation. One limitation to consider was the potential effect of adjusting face-to-face interviews to digital. Suggested improvements need to be considered for further eHRF development. Implication of research are on implementation barriers and facilitators within a feasibility study.

## Electronic supplementary material

Below is the link to the electronic supplementary material.

Supplementary Material 1

Supplementary Material 2

Supplementary Material 3

## Data Availability

Deidentified data may be available from the corresponding author upon request and subject to General Data Protection Regulation (GDPR) and the Swedish Ethical Review Authority requirements.

## List of abbreviations

ePRO: Electronic Patient-Reported outcome
PRO: Patient-Reported outcome
YHC: Youth Health Clinic
SEXIT: Sexual health Identification Tool
WHO: World Health Organization
eHRF: Electronic Health Report Form
IT- company: Information Technology company
NRS: Numerical Rating Scale
SUS: Satisfaction Usability Scale
SMS: short message service
Audit: Alcohol Use Disorder Identification Test
HBSC: Health Behavior in School-aged Children
GAD-7: General Anxiety Disorder 7-item scale
PHQ-9: Patient Health Questionnaire-9
SCOFF: acronym from the five questions in the SCOFF questionnaire
BSSS: Berlin Social Support Scale
GDPR: General Data Protection Regulation
