# Supplementary material for: Development and usability evaluation of an electronic health report form to assess health in young people: a mixed-methods approach
Source: BMC Med Inform Decis Mak. 2023 May 10;23:91. doi: 10.1186/s12911-023-02191-7 (PMC10170452; doi:10.1186/s12911-023-02191-7)
Supplement: Supplementary file 1 — Supplementary Material 1 [file 12911_2023_2191_MOESM1_ESM.docx]

# **Supplementary file 1**

## **eHRF prototype usability evaluation**

### **Qualitative data collection**

At the time for data collection, the YHC has a water damage and was partially closed. Also, due to the COVID-19 pandemic, regulations restricted real-life meetings, necessitating the interviews with young people to be digital. Hence, the interviews required the participants to have access to a smart phone, computer, or iPad. The participants gave digital (young people), written (healthcare professionals), or voice-recorded verbal (expert panel) consent before the interviews. Two of the young people were interviewed together and two individually. The healthcare professionals were interviewed individually. The healthcare manager participated digitally, and the expert panel participated in a digital group interview. Digital interviews were held via video meetings and face-to-face interviews were held at the YHC. The semi-structured interview guide was developed by the researchers, guided by the study aim. The guide was used for all participants. PVL performed the interviews, which lasted 50–90 minutes. All interviews were voice recorded with the hand-held Linear PCM Voice Recorder DDR-5300. All interviews were transcribed verbatim.

The interviews started with PVL registering the young participants in the IT company’s server (1), which granted access to a digital link, sent by SMS to their own smartphone, for access to the eHRF prototype health questions. When the participants opened the link, they were presented with a three-minute information film about the study and consent forms before they were asked to digitally consent to participate. The participants answered the eHRF prototype health questions, including background questions. They were then interviewed about their opinions of the usability of the eHRF prototype’s content and functionality.

At the end of the interviews, the young people, healthcare professionals the manager and the expert panel, answered the System Usability Scale questionnaire SUS). Those participating digitally were given the choice to respond orally to the statements in the SUS during the interview or e-mail the answers to PVL and all chose to answer orally during the interview. All participating digitally gave oral answers and PVL and ÅR took notes of their answers.

## **References**

1. Entermedic. Available from: https://www.entermedic.com/.

2. Brooke J. SUS: A "quick and dirty" usability scale. I: Jordan P, Thomas B, Weerdmeester B, McClelland I, redaktörer. Usability Evaluation in Industry. London: Taylor & Francis; 1996. p. 189-94.

3. Lewis JR. The System Usability Scale: Past, Present, and Future. International journal of human-computer interaction. 2018;34(7):577-90.

4. Göransson B. SUS Swedish. Rosenfeld media; 2001-2011. Available from: https://rosenfeldmedia.com/wp-content/uploads/2020/09/SUS-svensk.pdf.
