# Supplementary material for: Development and usability evaluation of an electronic health report form to assess health in young people: a mixed-methods approach
Source: BMC Med Inform Decis Mak. 2023 May 10;23:91. doi: 10.1186/s12911-023-02191-7 (PMC10170452; doi:10.1186/s12911-023-02191-7)
Supplement: Supplementary file 2 — Supplementary Material 2 [file 12911_2023_2191_MOESM2_ESM.docx]

## Supplementary file 2

## The electronic Health Response Form questionnaires; translations, references, and links in English

## Swedish questionnaires - translation into English

### SEXual health Identification Tool (SEXIT). No licence needed. Used in agreement with developer (spring 2020).

[SEXIT3.0EnglishSEXualhealthIdentificationTool (2).pdf](file:///C:\Users\1C28\Downloads\SEXIT3.0EnglishSEXualhealthIdentificationTool%20(2).pdf)

### Questions about your living habits: physical activity. No licence needed

[Frågor om dina levnadsvanor–Fysisk aktivitet–engelska (vgregion.se)](https://mellanarkiv-offentlig.vgregion.se/alfresco/s/archive/stream/public/v1/source/available/sofia/hs5940-679174602-438/native/Fr%C3%A5geformul%C3%A4r%20Fysisk%20aktivitet%2C%20Engelska.pdf)

### Questions about your living habits: tobacco. No licence needed

[Frågor om dina levnadsvanor–tobak–engelska (vgregion.se)](https://mellanarkiv-offentlig.vgregion.se/alfresco/s/archive/stream/public/v1/source/available/sofia/hs5940-679174602-441/native/Fr%C3%A5geformul%C3%A4r%20Tobak%2C%20Engelska.pdf)

### Questions about your living habits: eating habits. No licence needed

[Frågor om dina levnadsvanor–kost–engelska (vgregion.se)](https://mellanarkiv-offentlig.vgregion.se/alfresco/s/archive/stream/public/v1/source/available/sofia/hs5940-679174602-440/native/Fr%C3%A5geformul%C3%A4r%20Matvanor%2C%20Engelska.pdf)

Study-specific self-efficacy question for behavior change : *No licence needed*

“How confident do you feel to make a change to improve your health within this health area?”

## English questionnaires - links

### The Alcohol Use Disorders Identification Test-Concise (Audit-C) No licence needed

[Audit-C.pdf (anthc.org)](https://anthc.org/wp-content/uploads/2017/05/Audit-C.pdf)

### Generalized Anxiety Disorder 7-item (GAD-7) Scale No licence needed

[GAD-7.pdf (squarespace.com)](https://static1.squarespace.com/static/5b11943aaf20961cbb8f1315/t/5bb5131e24a6940b1fd4ef04/1538593567545/GAD-7.pdf)

### Patient Health Questionnaire (PHQ-9) No licence needed

[Patient Health Questionnaire (PHQ-9) (stanford.edu)](https://med.stanford.edu/fastlab/research/imapp/msrs/_jcr_content/main/accordion/accordion_content3/download_256324296/file.res/PHQ9%20id%20date%2008.03.pdf)

### The Scoff Questionnaire No licence needed

[Microsoft Word - SCOFF Questionnaire handout.doc (nutritionhealth.com.au)](https://www.nutritionhealth.com.au/site/assets/files/1064/scoff-questionnaire.pdf)

### Berlin Social Support Scales; sub-scales Need for support & Support seeking No licence needed

[berlin_social_support_scales_english_items_by_scale.pdf (midss.org)](https://www.midss.org/sites/default/files/berlin_social_support_scales_english_items_by_scale.pdf)

### The Health Behavior in School-Aged Children Symptom Checklist (HBSC) No licence needed

Stem question: How often during the last 6 months have you suffered from the following symptoms?

| Item | Symptom | Response options |
| --- | --- | --- |
| 1 | Headache | 1 Rarely or never  2 About every month  3 About every week  4 More than once a week  5 About every day |
| 2 | Abdominal pain |  |
| 3 | Back ache |  |
| 4 | Neck ache/shoulder ache |  |
| 5 | Dizziness |  |
| 6 | Sleeping difficulties |  |
