# Supplementary material for: Development and usability evaluation of an electronic health report form to assess health in young people: a mixed-methods approach
Source: BMC Med Inform Decis Mak. 2023 May 10;23:91. doi: 10.1186/s12911-023-02191-7 (PMC10170452; doi:10.1186/s12911-023-02191-7)
Supplement: Supplementary file 3 — Supplementary Material 3 [file 12911_2023_2191_MOESM3_ESM.docx]

# **Supplementary file 2**

## Results of the interviews

#### Captures overall health of young people but needs clarification

This category included the Electronic Health Report Form (eHRF) prototype content. It consists of the participants’ opinions on whether the eHRF prototype, with its health areas of mental, physical, and sexual health and social support, was found to capture the overall health of young people. However, there was room for change to improve the eHRF prototype for users. From this point, young participants, healthcare professionals, and the expert panel are included when the term “participants” is used.

#### Can identify health areas appropriate for young people

The participants were generally positive toward the eHRFs’ ability to identify health areas, appropriateness for young people, and health content. They agreed that the mix of questions contributed to capturing young people’s overall health.

… I thought you had most things... food, behavior, amount of exercise ... this much about physical and mental health. No, I think you had most of it. (Young person, 20 years old)

I think these are good areas. They are comprehensive and exactly what we talk about with young people today. (Healthcare Professional)

#### May make young people aware of the need for behavior change

The young participants believed that the eHRF prototype had the potential to make young people aware of the need for behavior change. They thought that it was usually difficult for young people to realize and admit that they had a health-related problem, and that they may not recognize the need to change their behavior to improve their health. Therefore, some believed that the self-efficacy question on behavior change could be difficult to answer. The healthcare professionals found these questions important, based on their experience of the challenge of having conversations about behavior change. The expert panel did not reflect extensively on this matter.

I think that we face this … for example with smoking… they [young people] say ‘no, no. no’, ‘they can stop when they want to’ or ‘there’s no problem with getting chlamydia ten times’ …they don’t think that it’s a problem and you never get anywhere… So, I think that’s a great question! (Healthcare Provider)

Although the health questions had the chance to increase awareness of the need for help, both young participants and healthcare professionals pointed out that some young people do not want help to change, or do not know how to perform a behavior change.

Maybe I don’t want to talk about that right now…. the anxiety, right now… I’m here for my birth control pills… (Young person, 16 years old)

#### Some questions and wordings irrelevant and difficult to understand

Some hesitation was expressed from the participants regarding the benefits of the eHRF prototype asking some sensitive and obvious questions, for instance about young people’s height and weight. Participants expressed that if necessary (e.g., for making medical decisions), these could be measured during the consultation.

Why do we want to know how much they weigh and how tall they are … it is quite sensitive for many … especially weight... (Expert panel)

The participants wanted a definition of “social support” and neutralization of gender descriptions, and they suggested changing “behavioral change” to “lifestyle change” and having more focus on family relations. The use of words uncommonly used by the younger population (e.g., the word “anxious”) risked causing confusion about the meaning of certain questions, and self-criticism. The problem was also considered by the healthcare professionals to be a matter for improvement.

It said “anxious” ... it was probably just me who did not understand the word, I think. (Young person, 16 years old)

In addition, the young participants responded positively to having several response possibilities that elicited more than a categorical yes or no response. The healthcare professionals were concerned that young people could perceive the health questions as extensive and hard to answer.

#### Fun, easy and optional will keep young people’s interest

The category describes the participants’ opinion that the design, layout, and structure of the eHRF prototype could help young people answer the health questions.

#### Needs an easy-going and neutral design

An easy-going and neutral design was considered important by the participants. The young YHC participants did not want to spend much more than 10 minutes answering the health questions in the eHRF prototype. They found the emojis refreshing and thought that they gave interest to the design and made the eHRF prototype look more fun. They suggested, however, that the emojis could possibly be exchanged for other pictures or even colors or fonts.

…refreshing and fun. There’s nothing negative with it… (Young person, 20 years old)

The expert panel and healthcare professionals, on the other hand, were hesitant or critical toward details in the layout, suggesting that the emojis used in the eHRF prototype could affect young people by portraying an emotion about the health area and questions it represented; they wanted the emojis to be changed to something more neutral, commenting that “less is more.”

… a smiley is so loaded with values – it shows a feeling … One may have a lot of problems with sexual health and there it is a figure with lots of hearts and a happy face … that it can be stressful ... (Healthcare Provider)

#### Must be able to choose to answer

Participants found it essential that young people could choose to answer the health questions before the meeting. Both young participants and healthcare professionals wanted young people to be able to disregard questions that they were uncomfortable answering.

But I think if it clearly states at the beginning that ‘if you are not comfortable answering some questions, then just click on “skip question”’.... (Young person, 18 years old)

The healthcare professionals reflected that if the opportunity to choose which questions to answer was only theoretic, it could make many YHC healthcare professionals hesitant to use the eHRF prototype.

... but there are also very many questions that you can’t [choose]. And then I think … if you start with this, as a youth, it is usually very difficult to say that “you know… I don’t want to continue”… it becomes a thought. And I also think that it can even cause staff to not want to hand out the eHRF (Healthcare Provider)

#### Potential contribution to improving the health consultation

This category includes the eHRF prototype’s contribution to health communication. It contains participants’ views on being able to honestly answer sensitive questions in the eHRF prototype. It also gives an idea of how the eHRF prototype could contribute to optimal use of the time at YHC.

#### May help to mediate sensitive health information

The participants had somewhat different opinions of the function of the eHRF prototype as a possible means to obtain information that may have been difficult to express verbally. The young participants found that responding to health questions before meeting with a healthcare provider could make it easier for them to answer hard questions honestly.

... instead of sitting there and trying to explain everything for half an hour, so you only have half an hour left of your time ... So, I thought it was good. (Young person, 16 years old)

However, the healthcare professionals found the questions were many and on very sensitive topics. They were unsure that a young person would answer them honestly or even complete the eHRF prototype questions. They also suspected that answering questions without knowing who they would meet at the YHC could make young people unwilling to use the eHRF prototype. The expert panel did not express any opinions on this matter.

It is quite extensive for young people to fill in, it feels like … it is very good support for us but there may be some who think it will be a little too much. (Healthcare Provider)

#### Helpful for planning and performing the meeting

The participants found that the eHRF prototype could be helpful before and during the meeting. The young participants believed that answering the health questions could make them more focused on their health and help them prioritize which health areas that were more important. The healthcare professionals agreed with the young participants and said that the eHRF prototype questions and the young people´s answers could form solid ground for talking with young people about health and contributing to their professional evaluation.

And so, you can .... you must go through this together for a while ... you look at this together. (Healthcare Provider)
